# Development of a Comprehensive Cough Therapy Program (CCTP) for chronic cough in India: a qualitative study

**DOI:** 10.1590/2317-1782/20242023347en

**Published:** 2024-10-11

**Authors:** Yamini Venkatraman, Vishak Acharya, Sindhu Kamath, Dhanshree Rajesh Gunjawate, Malavika Anakkathil Anil, Ajithesha Neriya Hegade, Radish Kumar Balasubramanium

**Affiliations:** 1 Department of Audiology and Speech Language Pathology, Kasturba Medical College Mangalore, Manipal Academy of Higher Education, Karnataka, Manipal, India.; 2 Department of Pulmonary Medicine, Kasturba Medical College Mangalore, Manipal Academy of Higher Education, Karnataka, Manipal, India.; 3 The MARCS Institute for Brain, Behavior and Development, Western Sydney University - Sydney, Australia.; 4 University College - Mangalore, Karnataka, India.

**Keywords:** Chronic Cough, Behavioral Treatment, Interprofessional Collaboration, Refractory, Qualitative Research

## Abstract

**Purpose:**

Chronic Cough (CC) is an emerging area of practice in speech language pathology. Behavioral treatment for managing CC has gained attention in the recent past. This study aimed to devise a comprehensive behavioural therapy program for CC by involving allied health professionals (AHPs), who are typically involved in management of CC.

**Methods:**

A qualitative methodology was used to devise a behavioral treatment module for CC. Practice patterns of medical professionals, AHPs and yoga practitioners for CC were gathered through semi-structured interviews. A constant comparative framework was used to recruit participants until data saturation was achieved. The interview transcripts were analyzed to identify relevant components for the module. A post-interview survey was conducted to finalize the module through a consensus-based approach.

**Results:**

Three themes and respective sub-themes were identified from analysis. The module was developed based on the treatment strategies followed by professionals and was labeled ‘Comprehensive Cough Therapy Program (CCTP)’. This comprised four components – similar to what is available in literature – patient education, breathing exercises, laryngeal hydration, and cough control strategies. This was devised using inputs from the interviews and evidence in the literature.

**Conclusion:**

In line with global guidelines, this behavioral treatment module can serve as a possible management option for CC.

## INTRODUCTION

A cough lasting over eight weeks is defined as ‘chronic cough’ (CC)^(1)^. A persistent cough of unidentified etiology despite extensive assessment or optimal treatment, is called an ‘unexplained chronic cough’ or ‘refractory chronic cough,’ respectively^(2,3)^. Such patients are primarily seen by pulmonologists and later by cough care specialists like allergists, laryngologists, or gastroenterologists to identify and treat underlying causes^(4)^. The medical line of treatment typically involves anti-asthmatic drugs (trials of inhaled corticosteroids), anti-acid drugs (proton pump inhibitors), and neuromodulatory agents (pregabalin, gabapentin, etc.). American College of Chest Physicians (CHEST) and the European Respiratory Society (ERS) provide guidelines for hierarchical prescription of the above-mentioned drugs^(2,3)^. These guidelines suggest a trial of non-pharmacological therapy, i.e., speech pathology/physiotherapy treatment, for the unexplained or refractory CC. However, speech language pathologists (SLPs) or physiotherapists (PTs) are not the primary points of contact, as patients often seek medical attention first^(5)^. This often leads to late referrals to allied health professionals (AHPs) from physicians after rigorous testing and treatment^(6,7)^. If we handle CC with a multi-, trans-, or inter-disciplinary approach, patients will have greater healthcare access and find quicker relief for their persistent cough.

As indicated, SLPs and PTs provide behavioral interventions for CC involving specific exercises and strategies. Literature has evidence for breathing retraining in improving symptoms of CC, either as a stand-alone regime^(8,9)^ or as part of speech pathology treatment^(10-12)^. The existing therapy programs include breathing training, relaxed breathing for cough suppression, and posture correction. However, these programs do not comprehensively address breathing. Appropriate breathing techniques may help deal with laryngospasms, vocal cord dysfunction, and other laryngeal etiologies commonly encountered with CC. It is also well known that breathing exercises specifically from pranayama can enhance lung capacity^(13)^ and, thus, aid while tackling bouts of cough.

Moreover, yoga is commonly practiced in India^(14)^, specifically pranayama in treating respiratory diseases, such as asthma and Chronic Obstructive Pulmonary Disease (COPD)^(15-19)^. There is an integration of ancient yoga into Western practices, and its benefits in improving lung functioning and capacity have been documented in literature^(17,18,20,21)^. The increasing global incorporation of yoga in everyday regimes and disease-specific treatment programs is an indication for considering yoga in the intervention of CC. Unsurprisingly, yoga, or rather its component, pranayama, can have a role in treating CC. We infused a component of ancient yoga, i.e., pranayama, into this module that might appease the Indian population and be better accepted. Thus, we aimed to devise a behavioral treatment module for CC that AHPs can provide.

In addition, this paved the way for considering an interprofessional collaboration in practice (IPCP) model for managing CC^(22)^. World Health Organization (WHO) considers IPCP to be a model that provides the highest quality of service by involving multiple professionals from various backgrounds^(23)^. We incorporated an inclusive approach by involving most stakeholders across disciplines to ensure comprehensiveness in patient care. In line with this, ERS and CHEST guidelines^(2,3)^ have included non-pharmacological therapies for CC, especially when the condition remains unidentified or refractory to medical treatment. Adapting from Western literature^(10-12)^ and practice guidelines^(2,3)^, behavioral treatment for CC was recently included in a consensus statement on managing cough in India^(24)^. Behavioral or non-pharmacological interventions for CC are emerging in Indian settings with the revised inclusive medical guidelines^(24)^.

To comply with and encourage the need for interprofessional collaborative practice in India, we invited healthcare professionals dealing with CC patients and gathered their practice patterns to develop this module. For this very purpose, we chose a qualitative methodology and conducted semi-structured interviews (SSIs) to collect relevant information on CC treatment practices, allowing flexibility in interviewing. The outcomes of this research aimed to develop a treatment module for CC which may improve interprofessional collaboration and, eventually the healthcare system^(22)^. These outcomes and aims align with the third sustainable developmental goal (SDG) adopted by the United Nations.

## METHOD

We aimed to devise a behavioral treatment module for CC using the constant comparative method (CCM) framework. The term behavioral treatment module will be used throughout this paper, and it refers to the intervention program with behavioral components developed specifically targeting CC. As an ideology of this framework, we conducted semi-structured interviews (SSIs) to collect practice patterns of professionals working with CC from multiple backgrounds. The findings have been reported using consolidated criteria for reporting qualitative studies (COREQ)^(25)^. The behavioral treatment module was devised by computing the analyzed information gathered through SSIs and existing evidence base. This study was approved by Kasturba Medical College Mangalore, Manipal Academy of Higher Education, Karnataka, Manipal (IECKMCMLR-11/2022/451).

### Study design and participants

In line with the CCM framework, SSIs were chosen to gather information from participants. The study participants were professionals from medicine, allied health, and yoga who worked with patients who had CC. Medical practitioners were involved to gain insights regarding their suggestions/guidelines recommended to the CC patients despite not practicing behavioral approaches.

Participants with a minimum clinical experience of five years in assessing/treating CC were recruited through purposive sampling. Table 1 depicts the details of the participants. Professionals from different institutions were approached by telephone and informed about the purpose of the study. They were asked for their willingness to participate in the interview. After obtaining their consent, they were contacted through e-mails with the interview details. Initially, we considered a minimum of two professionals from each discipline for the SSIs, but subsequently, more professionals were recruited. As this study employed a CCM approach, it allowed ongoing recruitment of participants to understand the practice patterns in CC better. With every completed interview, the data obtained was compared with the previous interview. If new inputs emerged, participants were further recruited until data saturation was achieved. If no new information emerged, participant recruitment was terminated for that field of discipline. None of the recruited participants refused to participate at any point in the study.

**Table 1:** The clinical experience (in years) and gender distribution of the participants

**Field of practice**	**Sub-specialty**	**n**	**Clinical experience (in years) (Mean±SD)**	**Gender distribution (Male/Female)**
Medical	Pulmonology	2	18.00±7.07	2/0
	Otorhinolaryngology	2	13.00±9.89	1/1
	General medicine	2	16.20±0.35	2/0
Allied health	Speech language pathology	3	6.00±1.32	0/3
	Physiotherapy	5	9.30±7.06	2/3
Yoga	Yoga	3	9.66±2.51	2/1
	**Total**	**17**	**11.05±6.22**	**9/8**

Caption: SD = Standard deviation

### Interview guide

The first author prepared interview guides based on existing literature on CC for the SSIs for each discipline, i.e., medical, allied health, and yoga groups. The interview guide consisted of introductory statements, key questions, transitory statements, probe questions, and closing statements (Appendix A). The focus of the interview was CC, specifically targeting the refractory, non-productive cough. Medical professionals deal with both productive and non-productive coughs; however, AHPs deal predominantly with the refractory type of CC. The key questions were grouped under these headings – 1) assessment of CC, 2) treatment of CC (a specific probe to check on awareness of behavioral intervention), and 3) suggestions for the module.

### Semi-structured interviews

The first author interviewed the participants face-to-face or online between March 2023 and May 2023. The interview mode was decided per the participant's convenience and scheduling logistics. The face-to-face interviews were conducted at the participant's workplace and did not include anyone other than the first author and the participant. The interviews were in English and audio-recorded using a digital voice recorder (Sony ICD-PX470) with the participant's consent. The online interviews were conducted, and audio-video recorded through the Microsoft Teams platform. The overall structure of the interview was as follows – greeting and welcome, a brief introduction to the purpose of the interview, key questions, follow-up/probe questions, and wrap-up. No repeat interviews were conducted for any participant.

The interview questions primarily focused on four broad areas – caseload, assessment, treatment, and suggestions for the module. Each broad area had three to four questions to its merit, and probe questions were asked by the interviewer to explore further. Initial questions were broad, and follow-up/probe questions were specific to target clinical assessment and treatment practices. The probes were used when responses were deemed inadequate or piqued the topic of interest or to clarify the participants’ statements. The participants’ responses were usually thorough and, sometimes, even answered more than one question targeted in the interview guide. Questions on intervention for CC were mainly to understand their practices and glean any possible suggestions from the participants for this treatment module. The recorded interviews were transcribed for further analysis.

### Transcription & analysis

The face-to-face interviews were transcribed using the online software^(26)^ for analysis. In comparison, interview transcripts were available from the Microsoft Teams platform for the online interviews. All transcripts were cross-checked and edited by the first author by comparing them with the recorded interviews for possible errors. After completing the quality check, the transcripts were analyzed. The content extracted from the transcripts was grouped into themes and sub-themes as per the interview guide's classifications. Suggestions given by the participants regarding the module were included to formulate the module.

### Post interview survey

Treatment strategies were identified and compiled from interview transcripts and evidence-based literature. This was collated and circulated to the participants through an online survey form. During the interview, the participants were informed regarding a post-interview survey. A specific question was formulated to decide the name for this developed module. Participants were given options to choose from and provide other suggestions as they saw fit. The survey responses were collated and were used in finalizing the components of the module using a consensus-based approach.

## RESULTS

### Data analysis

The primary purpose of the interviews was to obtain expert insights for the behavioral treatment module for CC. The duration of the interviews ranged between 25-60 minutes depending on the time taken by the respondents to answer questions, follow-up/probe questions, additional discussions, or any network-related glitches in case of online interviews. The analysis focused on identifying key components from the extensive transcripts. The aim was to filter out useful strategies and techniques for inclusion in the module. Relevant sections were identified as themes and sub-themes to understand participants’ knowledge and practice patterns with CC.

### Themes & sub-themes

Though we borrowed some aspects from the CCM framework for this qualitative research, we did not aim to build any new theories or code the data, as typically followed in research based on CCM and grounded theory. However, line-by-line analysis of transcripts was done to recognize broad themes and sub-themes, as the study focused on identifying key treatment strategies to develop the module. We identified three broad themes that aligned with the grouping followed in the interview guide: i) awareness & attitude, ii) assessment, and iii) treatment. Each of the themes had associated sub-themes under them, as discussed below.

#### Theme 1: Awareness & attitude

Participant’s thoughts on CC regarding awareness, the need for behavioral interventions, expanding the scope of AHPs, and multidisciplinary approach were grouped under this theme. This was deemed appropriate as participants’ knowledge and opinions laid the foundation for the interview.

Half of the medical professionals were aware of behavioral interventions for CC but had not recommended or referred it to their patients. Two medical professionals expressed interest in learning more about such interventions and were willing to recommend it to their patients in the future. Five medical professionals believed in the need for additional treatment rather than solely focusing on a medical approach. Some AHPs and yoga practitioners were aware and unaware of behavioral interventions for CC (Table 2). All SLPs and one PT were aware and actively engaged in providing behavioral interventions. The other four PTs mentioned that their caseload consisted majorly of patients with productive cough or paediatric population. Thus, their focus was facilitating sputum clearance/expectoration, not treating for non-productive cough. Two yoga practitioners reported having less experience with CC patients, but the other practitioner reported treating both productive and non-productive types of cough.

**Table 2:** Awareness of behavioral intervention among medical and allied health professionals

	**Medical professionals**	**Allied health professionals**	**Yoga practitioners**
**Behavioral treatment**			
Had awareness	0/6	4/8	0/3
Had heard of it	3/6	2/8	0/3
Had no awareness	3/6	2/8	3/3
**Thoughts on the need for a multidisciplinary module**			
Yes, the module will be helpful	6/6	8/8	3/3

The SLPs and PTs felt that CC, as a separate clinical entity, needs to be addressed in the curriculum at the under- or post-graduate level to build knowledge and exposure to such patients. When probed further, all participants agreed that more awareness and training were required. Regarding the resources for CC, the AHPs relied on Western tools and suggested the need for culturally/linguistically modified tools in India. Participants’ opinion on the need for developing a multidisciplinary behavioral treatment was gathered. All participants agreed on the need to develop such a program (Table 2) and expressed their views on its practical benefits (Appendix B).

#### Theme 2: Assessment patterns

All participants were involved in the assessment of patients with CC. They were asked to elaborate briefly on their assessment protocol for CC. Sub-themes identified in this section included information on referral sources, assessment procedures, and tools used by the participants.

The medical professionals reported that most of their CC caseload consisted of recurring patients, and the primary referral source was pulmonology. They referred and received patients from other disciplines, such as otorhinolaryngology and gastroenterology. Other need-based referrals included cardiology, psychology/psychiatry (if stress-related), and speech language pathology (for voice-related complaints). The AHPs identified medical professionals (in descending order of frequency of referrals received: pulmonology> otorhinolaryngology> internal medicine> general medicine) as their primary sources of referral for CC patients. The yoga practitioners indicated patient walk-ins and referrals significantly from Ayurvedic physicians and occasionally from medical professionals. All participants stressed the importance of taking a case history – to identify the onset, type, and duration of cough, cough triggers, diurnal vs nocturnal variations, lifestyle habits, and other related symptoms. All medical professionals and one SLP mentioned taking a drug history to identify cough-triggering drugs.

A stark difference was apparent in the assessment procedures used by the medical professionals and AHPs. The former used blood tests, radiographic imaging, pulmonary function tests, and invasive procedures like bronchoscopy, laryngoscopy, and endoscopy to diagnose CC. In contrast, the AHPs used subjective and patient-reported outcome measures to understand the symptoms and effects of CC on their quality of life. SLPs and PTs used cough symptom questionnaires, cough trigger checklists, quality-of-life questionnaires, and fatigue/exertion-related rating scales for their assessment. The PTs mentioned that there is no standard protocol for CC as their caseload consisted majorly of patients with productive coughs. The evaluation followed by yoga practitioners focused on identifying and diagnosing the underlying disease based on history, symptoms, physical exam, and yogic theories.

#### Theme 3: Treatment practices

The compiled opinions under this theme were of utmost interest, as they resonated with the aim of this study. All participants were involved in the treatment of cough. The literature on behavioral intervention for CC considers four components as fundamental, i.e., patient education & counseling, breathing exercises, laryngeal hydration, and cough control/suppression strategies. The recommendations received for the module were compiled under these four sub-themes. A general pattern observed among medical professionals was that they prescribed drugs to rule out the common causes of CC – upper airway cough syndrome, GERD, and asthma – and provided general dos and don’ts for managing cough triggers. AHPs and yoga practitioners focussed on techniques and strategies that entailed a behavioral change – postural, breathing, or cough control. An additional sub-theme, ‘complementary and alternative medicines,’ was also identified and included after the four sub-themes.

##### Sub-theme 1: Patient education & counseling

The two parts of this sub-theme addressed two different but essential aspects – 1) educating patients on cough and its effects and 2) counseling about the benefits of a behavioral approach in managing CC. The participants' perspectives compiled under this sub-theme were as follows. Patients need to be educated on the larynx (about where the cough occurs) and how to identify cough triggers. Helping patients understand the lack of underlying organic pathology and that they do not benefit from coughing becomes a crucial part of education. Participants emphasized avoiding forceful throat clearing or coughing whenever the patient experiences a mild itch/irritation to prevent it from becoming a habit. The participants frequently stated that CC could be anxiety-induced or stress-related and affect the patient’s quality of life and sleep. This makes it imperative to listen to patients’ symptoms and counsel accordingly on cough and its effects. Five participants indicated that they refer such patients to psychology. The medical professionals raised concerns about patient compliance in completing the prescribed dosage and appropriate usage of medical devices (such as inhalers).

##### Sub-theme 2: Breathing exercises

All AHPs, yoga practitioners and two medical professionals recommended breathing exercises. These were suggested as a relaxation strategy or to improve lung functioning and capacity. Relaxed throat breathing and pursed lip breathing were frequently suggested. Other techniques mentioned were the inspiratory breath hold manoeuvre and different types of pranayama. The techniques suggested by SLPs, PTs, and yoga practitioners had some degree of overlap in methodology.

##### Sub-theme 3: Laryngeal hygiene and hydration

All participants contributed to the bulk of this sub-theme. Apart from the medical line of treatment, most suggestions given by the medical professionals constituted ‘dos and don’ts’ in everyday life, which fell under this sub-theme. These suggestions aligned with the laryngeal hydration or vocal hygiene program usually recommended in voice and CC literature. Such measures targeted reducing laryngeal irritation and increasing hydration. This component included a combination of environmental, lifestyle, and diet modifications.

Environmental changes included identifying and staying away from triggers, taking precautions like wearing a mask when exposed to tussive stimuli (e.g., dust, pollens), and avoiding exposure to cold environments. Lifestyle modifications comprised stress/anxiety management (through meditation or deep breathing), weight reduction (physical exercises or yoga), and smoking (including passive)/tobacco/alcohol cessation. Dietary changes included frequent hydration and avoiding oily/spicy/junk food. Most participants insisted on avoiding cold food, environment, or beverages and consuming warm food/fluids instead. Postural advice included elevating the head position during sleep and avoiding lying down immediately after a meal to prevent acidic reflux.

##### Sub-theme 4: Cough control/suppression strategies

The literature proposes two ways of controlling cough: a suppression swallow or a breathing technique. AHPs significantly contributed to this component. They suggested the same two approaches available in the literature for cough suppression. A cough suppression swallow enables the patient to overcome the urge-to-cough sensation by performing a dry swallow. This is often accompanied by pressing hands together at chest level or doing a chin tuck to facilitate a ‘forced/tight’ swallowing gesture. The SLPs specifically mentioned looking out for any comorbidities or associated difficulties when performing a suppression swallow. The breathing technique, called ‘pursed lip breathing’, can control the urge-to-cough sensation and calm down the triggered laryngeal receptors. Additionally, SLPs recommended prescribing voice therapy exercises to address any vocal tension or hyperfunctioning of the laryngeal system, as these patients are often engaged in phonotraumatic behaviors, such as throat clearing and coughing.

#### Complementary and Alternative Medicines (CAM)

The participants advised to take appropriate vaccinations when indicated and against self-medication through over-the-counter drugs. Consuming home remedies or trying complementary and alternative medicines (CAM) for cough is common. The participants were enquired if their patients reported using CAM and how they aligned themselves towards recommending to patients. Their opinion statements are depicted in Table 3.

**Table 3:** Complementary and alternative medicines – the professional’s perspective

	**Medical professionals**	**Allied health professionals**	**Yoga practitioners**
Do patients report of using CAM?	Yes (6/6)	Yes (7/8)	Yes (3/3)
Do you recommend CAM to your patients?	No (6/6); with exception of steam inhalation and saltwater gargling	No (8/8)	Yes (3/3)
Opinion statements	→ *“Personally don’t recommend, take along with meds, if patients are comfortable”*	→ *“I think saying a direct no would not help”*	→ *“I don't tell any patient to stop medicines”*
			→ *“I suggest them to prepare a concoction - coriander jeera dry ginger powder and ajwain* (cumin)*”*
		→ *“We can identify if its helping them or not, and then maybe slowly give an explanation”*	
	→ *“Doctors themselves try these…”*	→ *“Not really (ask them to stop), unless something is a trigger for their cough. Only then… apart from that, no.”*	
	→ *“Their choice; advice about our specialty, that’s all”*		
		→ *“If they have a belief that it is helping them…Belief matters much more so...I don't try to change it”*	
	→ *“I don't advice”*		
	→ *“I do not prescribe..not part of my protocol*		
	→ *“Home remedies - continue if patients are comfortable”*		

### Preparing and finalizing the module through consensus

The final treatment module (Supplementary Material) was prepared based on existing literature and the inputs received from the participants during the SSIs. The cough suppression strategies given by the participants matched with existing literature. Techniques of pursed lip breathing and cough suppression swallow were included based on previously established studies on speech pathology management of CC^(12,27)^. When deciding the breathing exercises component, inputs from yoga practitioners were given more weightage - formulating instructions and dosage prescriptions - nonetheless, the dosages prescribed by SLPs and PTs aligned with the yoga practitioners. The compiled module was sent to all the participants through Google Forms survey (Appendix C). The participants rated the relevance of each component in the compiled module based on a 4-point rating scale (ranging between 1-4). The Google Forms survey link was mailed to all the participants, and they were asked to complete the survey in two weeks.

The results of the survey were compiled to devise the final module. The inclusion of a component in the module was decided based on the scores given by the participants for a given strategy/technique. The participants were asked to choose from options or suggest a suitable term for this module. Based on consensus, the most favored label for the module was Comprehensive Cough Therapy Program (CCTP). The strategies/techniques that were agreed upon by more than eight participants (with a score of 3 or 4), i.e., achieved consensus, were included in the final module. The suggestions given by the participants were considered and used to modify the techniques if necessary. Additionally, as a part of this module, handouts on environmental, postural, diet, and lifestyle-related changes were developed for patients. The components included in this module are quite similar to programs available in literature given by Vertigan et al.^(12)^ and TMCC^(27)^. However, the difference is in the collective input gathered from professionals across multiple disciplines and the extensive yoga based breathing related exercises (pranayama) that were incorporated.

## DISCUSSION

Devising a behavioral treatment module through the lens of a multidisciplinary team was the primary focus of this study. This was accomplished by conducting semi-structured interviews of healthcare professionals who treat CC. The information obtained from these interviews was collated under specific themes and sub-themes. The data was analyzed to identify possible treatment approaches – techniques and strategies – that will facilitate the amelioration of cough.

The awareness of behavioral management for CC among health professionals is still emerging. It was apparent from the responses analyzed that medical professionals did not send referrals to AHPs. Otolaryngologists and general medicine physicians referred their CC patients primarily to pulmonologists. Similar findings were reported in a study by Gowan et al.^(28)^. The delay or lack of referrals may be due to the lack of awareness or inhibitions in referring a behavioral intervention for CC^(7)^. However, all participants agreed on the need to devise a behavioral intervention for treating CC. The medical professionals welcomed the idea of a combined (i.e., behavioral and medical) approach for treating the refractory CC, as they had reported this to be a recurring clinical presentation. There may be many possible benefits of such a behavioral intervention, both from a patient’s and healthcare system’s perspective. It has the potential to enable timely care, reduce clinical visits, enhance quality of life, and facilitate a comprehensive intervention from one professional. Other favorable outcomes of this module might be improved interprofessional education, collaboration, early treatment initiation, and medical intervention.

There were similarities in CC practice trends among the professionals: the importance of taking a detailed case history, drug history, and counseling. Patients suffering from CC typically have cough triggers and/or a significant personal/medical history. If aspects of medical/personal history are missed during routine checks, it may lead to extensive testing, thus lengthening the whole process. Gowan et al.^(28)^ highlighted that not following guidelines could lead to delayed investigations and decreased quality of life in patients. Their study also documented that physicians relied on drug history, radiographic studies, laryngoscopy, and pulmonary function testing, which were in line with the responses from our participants. As for counseling, most participants suggested breathing-related exercises, avoiding cough triggers, lifestyle, and environmental changes.

These trends indicate that the practices of health professionals in CC are coinciding across few aspects of assessment and treatment. This is a positive finding as it indicates that basic concepts of behavioral intervention - reducing laryngeal irritation and identifying cough triggers - are targeted by even professionals unaware of cough-specific intervention. Though they did not follow a structured approach, their counseling covered considerable points from the four integral components of behavioral intervention, as mentioned in the previous section. Specifically, regarding treatment, medical professionals prescribed medications and provided recommendations on lifestyle and environmental changes. Their inputs for the treatment module predominantly comprised suggestions on do’s & don’ts to reduce cough. Thus, in the medical group, data saturation was achieved with fewer participants. However, more participants were recruited among AHPs and yoga practitioners as new information emerged with every interview. The inputs received from the SLPs, PTs, and yoga practitioners included breathing exercises, cough-related strategies, and lifestyle recommendations.

There was considerable overlap in the instructions and dosages given by AHPs and yoga practitioners for breathing exercises. Given the familiarity and common belief of yogic principles in breathing among physicians and people in India, breathing techniques based on yoga (pranayama) were included in the module. All participants recommended some form of breathing exercises, especially the pranayama based on yogic sciences, known for their general health benefits and positive effects on the respiratory system^(13,29,30)^. These increase lung capacity, respiratory functioning, and endurance^(30,31)^. SLPs/PTs highly recommended pursed lip breathing. Patients with CC are known to have associated respiratory or laryngeal dysfunctions like breathlessness, pain while breathing, laryngospasms, and hoarseness^(32-35)^. Participants also mentioned anxiety or stress as a triggering factor for the patient’s cough. Breathing exercises bring about relaxation while improving the pulmonary functioning of the patient. These exercises reduce anxiety or stress^(36)^, which are frequently associated in patients with CC^(32,37,38)^.

However, the cough suppression strategies were predominantly given by the SLPs and one physiotherapist only. Cough control or suppression strategies are based on principles of voice and swallowing management^(39)^. SLPs probably used these techniques because of their knowledge, exposure, and training in voice and swallowing. These techniques were suggested with slight modifications to adapt for cough suppression and depending on the patient’s abilities – for example, doing a cough suppression swallow with a chin tuck or while pressing the hands together tightly^(27)^. The effect of prior exposure to voice and swallowing-related caseload is well reflected in another component of the module, i.e., laryngeal hydration & vocal hygiene. This component had recommendations that directly targeted reducing cough by lowering throat irritation^(39,40)^. This is generally a familiar concept in managing cough, and most suggestions from PTs and yoga practitioners overlapped with those provided by SLPs.

Cough being a common symptom, patients attempt to alleviate their symptoms by taking home remedies or other medications, which is quite customary in India^(41)^. As expected, almost all participants reported that patients consumed some form of CAM. Most participants did not advise their patients on complementary medications. Yoga practitioners recommended home remedies or adjuvant Ayurvedic supplements. Patients also seek other alternative approaches such as homeopathy, acupuncture, and ayurveda, which are quite common in the treatment of CC in India^(42-46)^. Participants’ opinions were in sync with the well-being of their patients, as they understood that asking patients to discontinue their alternative practices would hinder the belief system. They intervened only when a particular remedy was triggering the patient’s cough but otherwise left it to their patient’s preferences.

‘Comprehensive Cough Therapy Program (CCTP)’ was the label chosen for the behavioral intervention module for CC as it received the highest consensus from the participants. A similar module termed Therapy Program for Management of Chronic Cough (TMCC) was developed by^(27)^, which focused on three components – counseling, cough suppression, and respiratory/laryngeal control – with a hierarchical prescription of strategies. A SLP or PT can provide CCTP as part of their routine clinical practice. The development of this behavioral intervention can be seen as a step towards holistic care and a way forward for expanding the scope of AHPs in treating CC. Referral patterns among medical professionals and AHPs exist, but they need to be expanded to CC as a standard practice to initiate the referral chain as early as the assessment stages. This will result in efficient healthcare management and better patient care, as envisioned by the United Nations as part of their third sustainable developmental goal.

### Limitations

The professionals who participated in the interviews were recruited based on their expertise in CC, which may have led to selection bias. However, the idea was to involve experts in the field of CC. Loss of data during online interviews due to internet fluctuations was a limitation. The first author clarified statements to ensure no data was missed; however, a slight possibility remains. Despite being qualitative research based on the CCM approach, we did not perform coding for analyzing transcripts or develop a new theory.

### Clinical implications and future considerations

This treatment module targeted CC of refractory origin. But it may be applied to coughs of other origins, especially long-COVID, where persistent cough is reported as common if not a frequent or a debilitating symptom ^(47,48)^. The intervention strategies in this module were derived from a multidisciplinary panel of participants working across different institutions, so these findings may be generalized across the given professionals recruited and work settings, which is a strength of our study. Literature has considerable evidence to show that components of CCTP can relieve CC symptoms. However, the efficacy of CCTP needs to be investigated in treating CC of all origins.

## CONCLUSION

Refractory or unexplained chronic cough is a specific phenotype for which treatment modalities are extensively researched on all fronts - drug therapies, clinical trials, and behavioral interventions. This study sought to develop a behavioral treatment module through semi-structured interviews with professionals in multiple disciplines. The outcome of this study is CCTP, which can be applied to patients with CC. Further studies examining the efficacy of CCTP are in progress.

## Appendix A The interview guide as used for conducting interviews for participants; some questions were modified based on the category of professional interviewed – medical vs allied health vs yoga

**Table:**

*Opening statement* – welcoming participant, briefing about the interview & recording consent	
**Introduction**	
Q1	Please tell me your name, years of experience specifically working with chronic cough.
*Transition statement*	
**Assessment**	
Q2	How many patients with chronic cough do you see in a month?
Q3	Are these patients’ new cases or recurring cases with same complaint?
Q4	Can you briefly elaborate on your assessment protocol?
Q5	What are some of the terms you use to diagnose this condition?
Q6	Involving other disciplines - Do you refer these patients to other professionals? Or do you work alone?
Q7	As an allied health professional, do you believe there is room for expansion of our scope of practice in chronic cough?
Q8	Are you the primary contact for the patients with CC or are they usually referred by medical professionals?
Q9	Do you feel you there are enough resources to assess such patients? Do you think you need more tools/resources/training?
*Transition statement*	
**Treatment**	
Q10	Do you provide treatment or refer to some other discipline for treatment?
Q11	What is your management protocol when treating these patients?
	Do you feel the medications/drugs provided as part of standard care resolves the cough complaints in all patients/all the time?
Q12	Are you aware of behavioral programs for CC? Do you think behavioral interventions from the field of speech language pathology and/or physiotherapy backgrounds can help?
Q13	Do your patients report on using CAM?
Q14	A combined approach to treatment in chronic cough by involving various disciplines – Pulmonology/ENT/SLP/PT. What are your thoughts on such an approach?
Q15	Do you think a comprehensive treatment module can be devised for chronic cough?
*Transition statement*	
**Treatment Module**	
**Information on available techniques in literature shared via PowerPoint presentation.**	
Q16	What additional techniques/strategies/suggestion from experience can be included, that you have seen to work in your patients? (from your discipline)
Q17	Can some guidelines on Do’s & Don’ts for such patients be prepared, as a part of this module?
Q18	What can this module be called – preferably with a non-discipline specific label?

## Appendix B Opinion statements of participants regarding the need and benefits of a multidisciplinary behavioral treatment for chronic cough

**Table:**

	Medical professionals	Allied health professionals	Yoga practitioners
*Needs assessment/ integration of the filed into the module*	→ *“I think you need a comprehensive thing to not just the medical line”*	→ *“We were not even aware that we did have a role in assessing and managing; the insight came from the pulmonologist”*	→ *“Mental thoughts…people get habituated, because of that they tend to get this type of issue* (refers to habitual cough). *So, we consider the patient as a whole...not just the physical body”*
	→ *“Time constraints in OPD”*	→ *“As a student, I was completely not aware of such a term even because the Rehabilitation Council of India, the syllabus prepared by them, we don't have anything like this”*	→ *“Any conflicts in the mind..As the yoga practice progresses, this gets reduced and whatever the impressions they had in their subconscious level, that gets relieved. Thereby we get a good result”*
	→ *“Practically not possible for a clinician to spend extra 10-15 minutes…We can definitely send those patients up and dedicated person will get them the therapy and call them for follow up”*	→ *“Most of the time we do not have much to offer...So at times it's like little being helpless. Of course, there are lozenges that we can advise, but it doesn't provide them a promising solution”*	
	→ *“Talking to patients - giving reassurance…doctors also need to take a moment to talk, not just prescribe medicines”*	→ *“Recently me or medical side, we have understood that this behavioral therapy works..got aware..speech therapy has got a role in the behavioral treatment…this awareness came in last five years”*	
		→ *“Definitely it is important that we expand into such an area… there is an increase in awareness of our own”*	
*Relevance of module*	→ *“Definitely (will help).. 110%...no doubt about that”*	→ *“I think it is very much necessary”*	→ *“Lifestyle has a very major role - healthy lives, like especially with the timings; try to modify; whatever you have* (the module) *that will work better”*
	→ *“Definitely this kind of module is going to be beneficial”*	→ *“I'm very happy that there is something like behavioral therapy for this kind of condition.”*	→ *“As the therapy goes..gradually after few months..the medicines are completely stopped”*
	→ *“Definitely will help…it should make a difference”*	→ *“Definitely it will work”*	→ *“Yoga is holistic; coordinate with the breath with the body movement”*
		→ *“Everybody wants the patient to be satisfied. If there is something that another professional can do, they (*medical professionals*) will definitely refer”*	
*Benefits of module*	→ *“Taking inputs from stakeholders… Learn from each other”*	→ *“AHP do spend longer duration of time with the patients. So probably that would be a good idea”*	→ *“Definitely it will work”*
	→ *“I think this will do good to the community… going to benefit a lot of specialties”*	→ *“The pulmonologist...noticing that the patients are really improving with our therapy…the symptoms are coming down…feel that they can refer more and more cases because the patients are reporting a better quality of life”*	
	→ *“Especially in allopathy we go on doing the same thing for everybody thinking that one-size-fits-all. When patient doesn't come back again, we take it for granted that the disease has been cured…in reality, patient might have adapted something else”*		

## Appendix C The rating form used for devising the final module.

Treatment Module Rating Form: Chronic Refractory Cough

Please rate the relevance of each component mentioned to be included in the treatment module. The rating system is as follows:

1 – the technique/strategy is not appropriate for the module at all

2 - the technique/strategy is somewhat appropriate for the module

3 - the technique/strategy is quite appropriate for the module

4 - the technique/strategy is highly appropriate for the module

**Table:**

	Treatment Components	Degree of relevance			
	**Name of the treatment module**				
	Behavioral Cough Therapy	1	2	3	4
	Cough Therapy Program	1	2	3	4
	Treatment module for long standing cough	1	2	3	4
	Multidisciplinary Cough Treatment	1	2	3	4
	Comprehensive Cough Therapy	1	2	3	4
	Comprehensive Cough Treatment	1	2	3	4
	***Please mention any suggestions/comments regarding the name of the module –***				
	**Component 1: Breathing exercises**	**Degree of relevance**			
1	Ensuring nasal rather than oral breathing	1	2	3	4
2	Concept of abdominal breathing – movement pattern & feedback	1	2	3	4
3	Relaxed deep breathing	1	2	3	4
4	Ujjayi Pranayama *(breath regulating technique indicated for respiratory issues)*	1	2	3	4
5	Bhramari Pranayama *(humming during exhalation for reducing stress/anxiety)*	1	2	3	4
6	Anulom Vilom Pranayama *(form of alternate nostril breathing)*	1	2	3	4
7	Sheetali pranayama *(breathing technique for cooling the body & mind)*	1	2	3	4
8	Inspiratory breath hold technique	1	2	3	4
	***Please mention any comments regarding Component 1 –***				
	**Component 2: Cough Suppression Strategies**	**Degree of relevance**			
9	Cough suppression swallow (just before the sensation of urge to cough)	1	2	3	4
	● Modifications available: Hand press/chin tuck (when not contraindicated)				
	Distraction techniques				
10	● Sipping water	1	2	3	4
11	● Sucking on sweet candies/lollies/lozenges	1	2	3	4
	Cough control techniques				
12	● Pursed Lip Breathing (with support)	1	2	3	4
13	● Pursed Lip Breathing (without support)	1	2	3	4
	***Please mention any comments regarding Component 2 –***				
	**Component 3: Vocal hydration & hygiene**	**Degree of relevance**			
14	Drinking water by taking as sips; 10-15 sips per hour (instead of gulps)	1	2	3	4
15	Steam inhalation (plain water) – by inhaling through mouth	1	2	3	4
16	Avoid smoking (active & passive)	1	2	3	4
17	Avoid alcohol consumption	1	2	3	4
18	Avoid caffeinated beverages (e.g. coffee, tea, cola)	1	2	3	4
19	Maintain a well-balanced diet	1	2	3	4
20	Avoid speaking loudly, yelling, cheering, or screaming	1	2	3	4
21	Avoid throat clearing/deliberate coughing	1	2	3	4
	***Please mention any comments regarding Component 3 –***				
	**Component 4: Patient education & counseling**	**Degree of relevance**			
22	To facilitate acceptance of a behavioral approach	1	2	3	4
23	Educate patients on the cough reflex, vicious cough cycle and cough reflex hypersensitivity/threshold	1	2	3	4
24	Explain the negative effects of repeated coughing	1	2	3	4
25	Explain the benefits of cough suppression – reduced cough symptoms & better quality of life	1	2	3	4
26	Educate patients on voluntary control of cough - internalisation of control over their cough	1	2	3	4
	***Please mention any comments regarding Component 4 –***				
**Additional techniques if some vocal pathology (laryngospasm/vocal fold paresis) is suspected**					
27	Vocal adduction exercises				
28	Breath hold – cough – phonate				
29	Power adduction exercises with /i/				
***Please mention your comments/suggestions, if any -***					
**Relaxation exercises to reduce laryngeal hyperfunction if present**					
30	Head & neck stretches				
31	Straw phonation				
32	Trills – bilabial & tongue				
33	Humming				
34	/u/-buzz				
35	Gentle throat massage				
***Please mention your comments/suggestions, if any –***					

## Supplementary Material

Supplementary material accompanies this paper.

Comprehensive Cough Therapy Program

This material is available as part of the online article from https://doi.org/10.1590/2317-1782/20242023347en
