# Supplementary material for: Development of a Comprehensive Cough Therapy Program (CCTP) for chronic cough in India: a qualitative study
Source: Codas. 2024 Oct 11;36(6):e20230347. doi: 10.1590/2317-1782/20242023347en (PMC11529996; doi:10.1590/2317-1782/20242023347en)
Supplement: Comprehensive Cough Therapy Program [file codas-36-6-e20230347-Suppl.pdf]

Comprehensive Cough Therapy Program

The Comprehensive Cough Therapy Program was developed as part of a PhD thesis. This module targets patients with a complaint of long-standing cough, i.e., chronic cough. This was devised based on existing literature and inputs from professionals across various backgrounds.

Component 1: Patient education & counselling
• Educate on the laryngeal structures involved in cough & the impact of the phonotrauma on larynx<br>• Facilitate better understanding of triggers and help them identify from their environment<br>• Educate on urge-to-cough sensation, cough reflex and cough reflex hypersensitivity<br>• Educate patients on the vicious cough cycle and the negative effects of repeated coughing<br>• Facilitate acceptance of a behavioural approach<br>• Explain the benefits of cough suppression – reduced cough symptoms & better quality of life<br>• Educate patients on voluntary control of cough - internalisation of control over their cough

Component 2: Vocal hygiene
**Do’s**
• **Environmental changes**:<br>○ Identify things that trigger your cough - incense sticks, mosquito repellents, perfumes, dust, pet animals<br>○ Stay away from your triggers initially, until you learn how to control your cough sensation<br>○ Wear a mask while dusting and cleaning at home<br>• **Diet changes**:<br>○ Eat a well-balanced diet every meal<br>○ Do not skip meals and eat when you feel hungry!<br>○ Hydrate yourself. Drink plenty of water.<br>○ Drink water in small sips (not gulps) through the day<br>• **Lifestyle changes**:<br>○ Steam inhalation through your mouth<br>○ Engage in some kind of physical activity– exercises/yoga/walking<br>○ Engage in meditation and deep breathing techniques to reduce any stress<br>○ Focus on weight reduction in a healthy manner, if needed<br>• **Postural changes**:<br>○ If you are experiencing reflux/heartburn during sleep, shift to a slightly elevated/upright position<br>○ Use pillows to prop up your head<br>• Take vaccinations – if indicated for allergies, as per physician’s guidance<br>• Consult your physician if your symptoms are not improving

Don’ts’s
- Do not self-medicate by buying medicines from pharmacy without a doctor’s prescription   **Environmental changes**:   - Avoid/reduce exposure to cold environment   - Do not sleep directly under the fan/AC draft   - Avoid exposure to dusty/smoky areas (construction sites, cleaning areas)   **Diet changes**:   - Avoid/reduce oily and spicy, fatty foods, junk food   - Avoid/reduce cold food – chilled water, cold drinks, ice-creams, etc.   - Avoid caffeinated drinks and beverages   **Lifestyle changes**:   - Avoid intake of alcohol   - Avoid smoking and chewing tobacco   - Avoid passive/second-hand smoking   - Avoid wearing tight clothes   - Do not sleep or lie down immediately after a meal

Component 3: Breathing exercises<br>*(All exercises are not mandatory - exercises will be chosen based on patient's performance)*			
	**Items**	**Instructions/Steps**	**Dosage**
1	Ensuring nasal rather than oral breathing<br><br>**<u>Rationale –</u>**<br>• To reduce the drying effect of mouth breathing<br>• To reduce laryngeal irritation	To be followed at rest and during speech activities	
2	Ensuring that patient engages in abdominal breathing pattern<br><br>**<u>Rationale –</u>**<br>• To modify dysfunctional breathing pattern	• When taking in a deep breath, feel your lungs and abdomen expanding outwards<br>• When breathing out, observe the emptying of lungs and inward movement of your abdomen<br>• Can take proprioceptive feedback by placing hands on chest and abdomen<br>• Feedback to be given until the concept is mastered	
3	Ujjayi Pranayama<br><br>**<u>Purpose –</u>**	• Please be seated comfortably (preferably cross-legged posture)<br>• Take a deep breath slowly through your nose and exhale slowly through your nose, as long as you can	21 cycles per set; twice a day

	• This helps in expansion of the lungs, provides endurance and improves focus on breath	• Take hand support for tactile feedback to feel the abdominal movements, if needed	
4	Relaxed throat breathing<br><br>**<u>Rationale –</u>**<br>• To modify dysfunctional breathing pattern<br>• To reduce laryngeal muscle tension around neck & upper chest area	• Please be seated comfortably (preferably cross-legged posture)<br>• Take a deep breath through your nose and exhale slowly with a relaxed and slightly expanded throat (as for a yawn)<br>• Do not force the air in or out, gently and slowly breathe every cycle, without any shoulder elevation or tension in the neck area.<br>• Take hand support for tactile feedback, to feel the abdominal movements, if needed	21 cycles per set; twice a day
5	Bhramari Pranayama<br><br>**<u>Purpose –</u>**<br>• Facilitates relaxation & improves concentration	• Please be seated comfortably (preferably cross-legged posture)<br>• Take a deep breath slowly through your nose and on exhalation, produce a humming sound for as long as you can (like saying ‘hmmmmm’)	12 cycles per set; twice a day

6	Anulom-Vilom Pranayama<br><br>**<u>Purpose –</u>**<br>• Cleansing of the respiratory system<br>• Helps in purification the nerves	• Please be seated comfortably (preferably cross-legged posture)<br>• Close your right nostril with your thumb and inhale through your left nostril<br>• Now, close your left nostril with your ring and little finger and exhale through your right nostril<br>• After completely breathing out, inhale through your right nostril<br>• Now, close your right nostril and exhale through your left nostril<br>• This is one cycle	21 cycles per set; twice a day<br><br>**<u>Note:</u>** After every exhalation, remember to breathe in from the same nostril from which you exhaled.
7	Sitali pranayama<br>*(This can cause dryness; can be used if it doesn't trigger cough in patients)*<br><br>Sitkari pranayama<br>*(If patient cannot roll the tongue, this variation will be used)*<br><br>**<u>Purpose –</u>**	• Please be seated comfortably (preferably cross-legged posture)<br>• Inhale through a curled tongue and exhale through the nose<br>• Swallow now if the throat feels dry<br>• If unable to curl the tongue - Inhale through the teeth, with the lips parted and the tongue floating just behind the teeth	12 cycles per set; twice a day

	• Cooling exercises to calm the mind and regulate cardiorespiratory rate		
8	Jalandhara bandha (Inspiratory breath hold technique)<br><br>**<u>Purpose –</u>**<br>• To facilitate breath retention for a long period<br>• Provides a stretch for the neck region	• Please be seated comfortably & keep your back straight<br>• Keep your chest & shoulders relaxed<br>• Closing your eyes, breathe in deeply and retain the breath<br>• Now, lower your head towards your chest and press your chin firmly<br>• Hold your breath for as long as you are comfortable<br>• Slowly raise your head and exhale gently	10 cycles per set; twice a day
9	Savasana/Yoga Nidra<br><br>**<u>Rationale –</u>**<br>• For complete relaxation of the mind and the body	• Lie flat on your back without any pillow and close your eyes.<br>• Keep your legs a little apart and let your feet and knees relax completely, toes facing to the sides.	Practice this immediately after the breathing exercises for 5 to 10 minutes

	• Facilitates reduction of stress and better sleep quality	• Place your arms alongside, yet a little spread apart from your body; keep your palms open, facing upward<br>• Keep breathing slowly, gently, deeply and allow your breath to relax you more and more	

Component 4: Cough control techniques<br>→ These strategies can be practiced by the patient whenever they feel the urge-to-cough (UTC) sensation or need to clear their throat<br>→ These can be used when they are exposed to triggers (in clinical or real-life scenarios)<br>→ All strategies will be taught to the patient – they can choose to engage in any technique which provides them maximum ease and comfort of use<br>→		
**Items**	**Instructions/Steps**	**Dosage**
Cough suppression swallow<br><br>**Rationale –**<br>• To identify the UTC and voluntarily suppress or substitute it to overcome the cough<br>• This helps in breaking the vicious cycle of cough	• The patient will be asked to identify the urge-to-cough sensation (usually a tickle, an itch or an irritation)<br>• Once the urge is identified, it can be overcome by either:<br>→ An effortful swallow, while pressing the hands together<br>→ An effortful swallow while bending down towards the chest<br>→ An effortful swallow<br>This can be practiced when there is a sensation to clear the throat and during the cough as well	Whenever the patient is experiencing UTC or the feeling to clear the throat

Distraction techniques<br><br>**<u>Rationale –</u>**<br>• To identify the UTC and voluntarily suppress or substitute it to overcome the cough<br>• This helps in breaking the vicious cycle<br>• Also provides some laryngeal hydration	• The patient will be helped to identify the urge-to-cough sensation (usually a tickle, an itch or an irritation)<br>• Once the urge is identified, it can be overcome by taking a few sips of water (room temperature/lukewarm)<br>• Sucking on sweet candies/lollies/ lozenges can be another alternative to overcome UTC	Whenever the patient is experiencing UTC or the feeling to clear the throat
Cough control through breathing<br>(Pursed Lip Breathing)<br><br>**<u>Rationale –</u>**<br>• To identify the UTC and voluntarily suppress or substitute it to overcome the cough<br>• This helps in breaking the vicious cycle	• This can be practiced when there's a sensation to clear the throat and during the cough as well<br>• Take a deep breath slowly through your nose<br>• For exhalation, pucker your mouth, as if drinking through a straw<br>• Slowly exhale through your partly opened mouth<br>• This can be carried out as long as necessary to overcome the urge or cough	Whenever the patient is experiencing UTC or the feeling to clear the throat

• To maximize expiratory flow and reduce respiratory rate	• If needed, the patient can be asked to take support by leaning forward and placing their hands on a table<br>• Take hand support for tactile feedback, if needed	

Additional component: Voice therapy techniques<br>*(These exercises can be prescribed in case of any associated vocal hyperfunction)*	
**Items**	**Description**
**Yawn sigh**	• To imitate a yawn to expand the pharynx and to stretch and relax the extrinsic laryngeal muscles<br>• Following the yawn, a gentle exhalation with a light phonation - prolonged, easy, open-mouthed exhalation after yawn<br>• Once the yawn–phonation is achieved, to say words beginning with /h/ or with open-mouthed vowels
**Vocal function exercises**	• Sustaining the /i/ vowel for as long as possible on a musical note (F) above middle (C) for females and boys, (F) below middle (C) for adult males.<br>• Gliding from your lowest note to your highest note on the word ‘‘knoll.’’<br>• Gliding from your highest note to your lowest note on the word ‘‘knoll.’’<br>• Sustaining the musical notes (C-D-E-F-G) for as long as possible on the word ‘‘knoll’’ minus the ‘‘kn.’’
**Laryngeal Massage**	• Encircle the hyoid bone with the thumb and middle finger. Work back posteriorly until the major horns are felt.<br>• Apply light pressure with the fingers in a circular motion over the tips of the hyoid bone.<br>• Repeat this procedure with the fingers from the thyroid notch, working posteriorly.<br>• Find the posterior borders of the thyroid cartilage (medial to the sternocleidomastoid muscles) and repeat the procedure.

	• With the fingers over the superior borders of the thyroid cartilage, begin to work the larynx gently downward and laterally at times.
**Lip & tongue trills**	• Begin by sustaining a trill on a comfortable pitch note<br>• Modification of task – ascending and descending glides
**Resonant voice/ Forward focus**	• Using easy voice associated with vibratory sensations in facial bones<br>• Use of nasal consonants (“m,” “n,” and “ng”) to facilitate training of the vibratory “resonance”.<br>• Basic training gesture – ‘Holm-molm-molm…’<br>**Stages** – all voiced → voiced-voiceless → any phrase →paragraph → conversation
**Humming**	Humming with the lips closed or nearly closed while producing with a forward focus
**Head & neck stretches**	These exercises can be given in case of physical strain or tension around the neck and shoulders - a sequence of:<br>• rotation of the shoulders backwards and forwards<br>• movement of head flexion and extension<br>• rotation of the head to the left and right and vice versa
